# Highly Stable, Flexible, Transparent Hybrid Strontium Titanate Conductive Thin Films with Embedded Cu Nanowires

**DOI:** 10.3390/ma18102398

**Published:** 2025-05-21

**Authors:** Ming Liu, Shihui Yu, Lijun Song, Jiesong Li, Jian Feng

**Affiliations:** 1 School of Materials Science and Engineering, Luoyang Institute of Science and Technology, Luoyang 471023, China; liumingming45@163.com; 2 School of Power Electrical Engineering, Luoyang Institute of Science and Technology, Luoyang 471023, China; lijunsong1983@126.com; 3 Triumph Information Display Materials (Luoyang) Co., Ltd., Luoyang 471023, China; 15005520311@163.com; 4 Luobo Group Luoyang Longhai Electronic Glass Co., Ltd., Luoyang 471023, China; 13838869907@163.com

**Keywords:** transparent conductive, thin films, flexible, Cu NWs

## Abstract

To meet the stringent demands of next-generation flexible optoelectronic devices, a novel fabrication approach is employed that integrates the spray-coating of copper nanowires (Cu NWs) with the magnetron sputtering of SrTiO_3_ thin films, thereby yielding SrTiO_3_/Cu NWs/SrTiO_3_ hybrid thin films. The incorporation of the SrTiO_3_ layers results in improved optical performance, with the transmittance of the Cu NW network increasing from 83.5% to 84.2% and a concurrent reduction in sheet resistance from 16.9 Ω/sq to 14.5 Ω/sq. Moreover, after subjecting the hybrid thin films to 100 repeated tape-peeling tests and 2000 bending cycles with a bending radius of 5.0 mm, the resistance remains essentially unchanged, which underscores the films’ exceptional mechanical flexibility and robust adhesion. Additionally, the hybrid thin films are subjected to rigorous high-temperature, high-humidity, and oxidative conditions, where the resistance exhibits outstanding stability. These results substantiate the potential of the SrTiO_3_/Cu NWs/SrTiO_3_ hybrid thin films for integration into flexible and wearable electronic devices, delivering enhanced optoelectronic performance and long-term reliability under demanding conditions.

## 1. Introduction

Flexible transparent conductive thin films (TCFs) are the essential components in modern electronics, finding applications in devices such as organic light-emitting diodes, touch screens, wearable sensors, solar cells, and transparent heaters [1,2,3,4,5]. Indium tin oxide (ITO) is widely employed in TCFs due to the exceptional optical transmittance and low sheet resistance [6,7]. However, ITO faces significant challenges in flexible electronics because of the high cost and limited availability of indium, as well as the inherent brittleness [8]. Consequently, there is growing interest in exploring alternative materials to replace ITO [9,10,11]. Metallic nanowires (NWs) have emerged as a promising candidate due to their excellent electrical conductivity, mechanical flexibility, and transparency [12,13,14,15]. Although gold and silver NWs deliver outstanding performance, their high cost precludes their practical application. In contrast, Cu NWs have attracted considerable attention due to their low cost [15,16]. However, the high susceptibility to oxidation when exposed to ambient conditions undermines the long-term durability [17]. Moreover, when Cu NWs are deposited directly onto polymer substrates, the poor adhesion often leads to detachment, bending, or peeling [18]. These drawbacks significantly impede the widespread adoption of Cu NWs in flexible TCFs.

A number of approaches have been investigated to enhance the oxidation resistance of Cu NWs [19,20]. Passivation layers composed of highly stable metals such as Au [21], Pt [22], Ni [23], and Ag [24] have been studied. However, controlling the precise thickness of these metallic coatings remains difficult, often resulting in diminished optical transmittance and failing to address the inherent adhesion challenges of Cu NWs. Another commonly explored strategy is the deposition of graphene layers onto Cu NW networks to serve as a protective barrier. Nevertheless, the intrinsic defects present in thin graphene coatings offer inadequate protection, and while thicker graphene layers can improve stability, they concurrently cause a significant reduction in optical transmittance [25]. In contrast, several metal oxides—including Al-doped ZnO [26], Sb-doped SnO_2_ [27], Al_2_O_3_ [28], and TiO_2_ [29]—have been reported to effectively mitigate the oxidation of Cu NWs. These oxide coatings not only enhance the durability and adhesion of Cu NW networks but also maintain the optical and electrical performance of the thin films, rendering them a more promising solution for improving the overall stability of Cu NW-based transparent conductive films.

Strontium titanate (SrTiO_3_), as a typical perovskite-type wide-bandgap semiconductor material (with a room-temperature bandgap of approximately 3.2 eV) [30], has demonstrated significant application value in photodetectors, capacitor dielectric layers, and photocatalytic materials due to its superior optical transmittance (>80% in the visible-light region) and unique dielectric properties [30,31]. However, research on the composite design of strontium titanate thin films with copper nanowires (Cu NWs) to achieve synergistic enhancement in optoelectronic properties through interface engineering remains unexplored in the literature. In this study, flexible transparent conductive thin films composed of the SrTiO_3_ and Cu NWs are fabricated via RF magnetron sputtering combined with spray-coating. The pristine Cu NW networks exhibit a sheet resistance of 16.9 Ω/sq at a transmittance of 83.5%. After the combination of SrTiO_3_ thin films, the sheet resistance is decreased to 14.5 Ω/sq, while the transmittance is increased to 84.2%. Moreover, the SrTiO_3_/Cu NWs/SrTiO_3_ hybrid thin films retain the mechanical flexibility and resistance stability under high temperature, high humidity, and severe oxidation.

## 2. Materials and Methods

SrTiO_3_/Cu NWs/SrTiO_3_ hybrid thin films were deposited onto transparent polyethylene terephthalate (PET) substrates via a combined RF sputtering and spray-coating approach. In this process, both the top and bottom SrTiO_3_ layers—each 50 nm thick—were fabricated using SrTiO_3_ ceramic targets of 99.99% purity. The ceramic targets, with dimensions of 50 mm in diameter and 5 mm in thickness, were sputtered in an environment of 99.999% high-purity argon at a controlled pressure of 1.0 Pa and a sputtering power of 60 W, while the substrate temperature was maintained at room temperature throughout deposition. Subsequently, an optimized spray-coating technique was employed to uniformly deposit the Cu NW solution onto the PET substrate, ensuring consistent coverage. The spraying duration was 5 s. After drying at 60 °C, the next coat was applied. The process was repeated one to six times. The coated substrate then underwent an acid leaching treatment using a 70% acid solution for one minute, effectively removing residual organic contaminants from the film surface. Finally, the Cu NW networks were carefully extracted from the solution and dried under a stream of nitrogen gas, thereby ensuring complete moisture removal and promoting film stabilization.

The film structure was characterized using a D/MAX-RB X-ray diffraction instrument (XRD, Rigaku, Japan). The surface morphology was examined with a field emission scanning electron microscope (FE-SEM JSM-7001F, JEOL, Japan) and atomic force microscopy (AFM, Bruker Multimode 8, USA). Electrical properties were measured using an SX1934 four-probe resistance tester (BAISHEN, Suzhou, China), and optical properties were determined via a Varian Cary 5000 ultraviolet–visible–near-infrared spectrometer (Agilent, USA). Additionally, the film’s flexibility was evaluated using a Nanoupe+ flexible tester (NANOUP ELECTRONICS, Changsha, China).

## 3. Discussion

**Figure 1:**
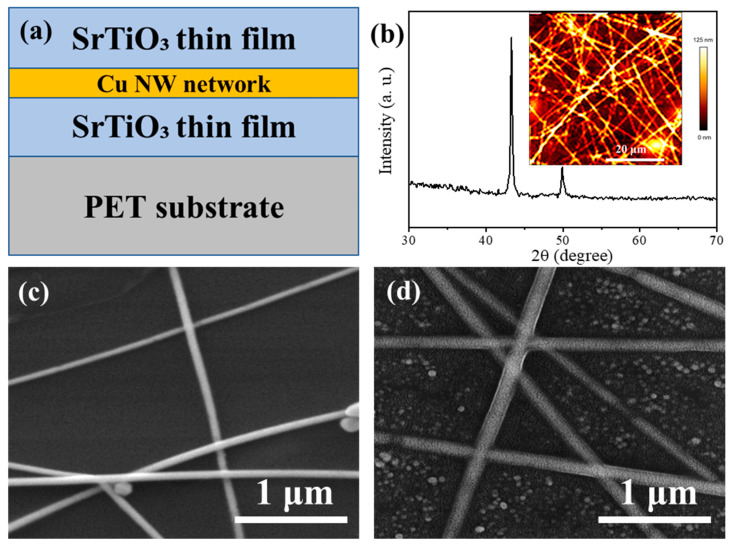
(**a**) The schematic structure of the SrTiO_3_/Cu NWs/SrTiO_3_ hybrid thin films deposited on PET substrates; (**b**) the XRD patterns of the SrTiO_3_/Cu NWs/SrTiO_3_ hybrid thin films, where the inset shows the AFM image of SrTiO_3_/Cu NWs/SrTiO_3_ hybrid thin films. The FE-SEM image of the (**c**) Cu NW networks and (**d**) SrTiO_3_/Cu NWs/SrTiO_3_ hybrid thin films.

Figure 1a presents a schematic structure of the SrTiO_3_/Cu NWs/SrTiO_3_ hybrid thin films deposited on PET substrates. In this configuration, the Cu NW network is encapsulated between two sputtered SrTiO_3_ layers, with the sheet resistance and transmittance being strongly dependent on the Cu NW density. The nanowire density is controlled by varying the number of spray-coating cycles used to deposit the Cu NW dispersion at a fixed concentration. The SrTiO_3_ thin films serve to encapsulate the Cu NWs effectively, thereby substantially improving their stability. Notably, these SrTiO_3_ thin films exhibit strong adhesion to the PET substrate [32], which further reinforces the attachment of the Cu NW network and ensures the composite maintains its structural integrity during subsequent processing and application. Figure 1b displays the XRD patterns of the SrTiO_3_/Cu NWs/SrTiO_3_ hybrid thin films, revealing two prominent diffraction peaks at approximately 43.2° and 50.1°. These peaks correspond to the (1 1 1) and (2 0 0) planes of pure face-centered cubic copper, with the Cu NWs demonstrating a preferential orientation along the (1 1 1) plane [33]. No characteristic diffraction peaks for SrTiO_3_ are detected, because the films, deposited at room temperature, remain amorphous; the crystallization of SrTiO_3_ typically requires temperatures above 500 °C [34]. The inset of Figure 1b shows an AFM image of the SrTiO_3_/Cu NWs/SrTiO_3_ hybrid thin film. The Cu nanowires are evenly distributed, but their interwoven structure raises the film’s surface roughness to 123 nm. Figure 1c shows an FE-SEM image of the Cu NW networks. The nanowires are randomly interwoven and uniformly distributed, thereby forming a percolating network that underpins the film’s favorable optical and electrical properties. Figure 1d further reveals the surface morphology of the SrTiO_3_ thin films, where the Cu NW junctions appear more tightly connected, indicating enhanced inter-nanowire contact and improved mechanical robustness. Importantly, the overall distribution of the Cu NW network is preserved following the deposition of the top SrTiO_3_ thin films, suggesting that the sputtering process does not adversely alter the underlying nanowire architecture.

**Figure 2:**
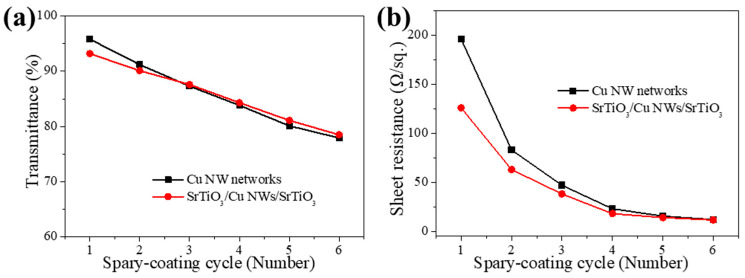
(**a**) Optical transmittance measurements and (**b**) sheet resistance for Cu NW networks and SrTiO_3_/Cu NWs/SrTiO_3_ hybrid thin films fabricated with varying numbers of spray-coating cycles.

Figure 2a presents the optical transmittance measurements for both Cu NW networks and SrTiO_3_/Cu NWs/SrTiO_3_ hybrid thin films fabricated with varying numbers of spray-coating cycles. Following a single spray-coating cycle, both types of samples exhibit an optical transmittance of above 90%. With additional cycles, the Cu NW density increases, leading to a gradual decline in transmittance. After five cycles, the transmittance for both samples decreases to below 80%. At such high nanowire densities, the Cu NW networks become sufficiently dense and thick, effectively acting as a reflective mirror. This observation underscores the importance of precisely controlling the Cu NW density to optimize the optical properties of SrTiO_3_/Cu NWs/SrTiO_3_ hybrid thin films when they are employed as top window electrodes. Notably, under identical spray-coating conditions, the SrTiO_3_/Cu NWs/SrTiO_3_ hybrid thin films demonstrate a slightly higher transmittance compared to the pristine Cu NW networks. This improvement stems from the SrTiO_3_ layer, which tightens the junctions between the Cu nanowires, smooths the film surface, and therefore reduces optical scattering.

Figure 2b depicts the evolution of sheet resistance as a function of spray-coating cycles for the samples. With increasing spray-coating cycles, the enhanced density of the Cu NW networks leads to the formation of additional electron transport pathways, thereby progressively reducing the sheet resistance [28]. Moreover, the incorporation of SrTiO_3_ thin films contributes to a further modest decrease in sheet resistance. As evidenced in Figure 1d, the deposition of SrTiO_3_ induces tighter interconnections at the junctions between individual Cu NWs, likely due to the impact of SrTiO_3_ particles during the deposition process, which in turn improves the contact between the nanowires and reduces inter-junction resistance [35]. Consequently, the overall sheet resistance of the SrTiO_3_/Cu NWs/SrTiO_3_ hybrid thin films is lower compared to that of the bare Cu NW networks.

**Figure 3:**
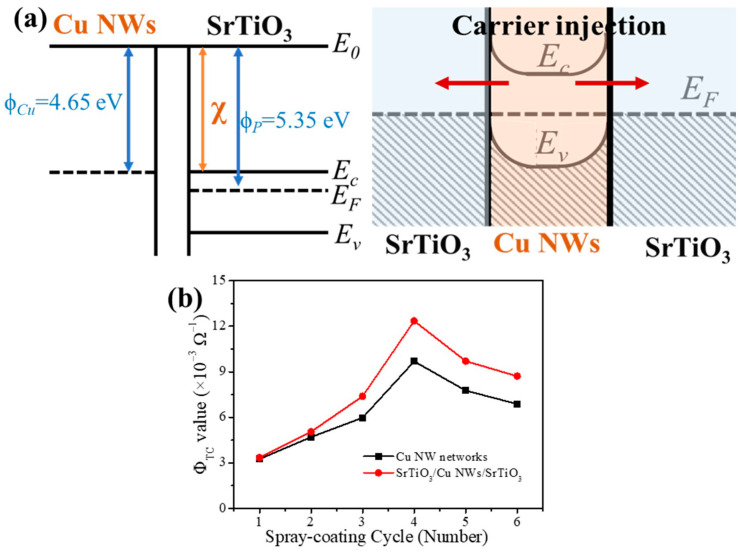
(**a**) Schematic band diagram for the SrTiO_3_/Cu NW interface. (**b**) Calculated *Φ_TC_* value for the Cu NW networks and SrTiO_3_/Cu NWs/SrTiO_3_ hybrid thin films with different spray-coating cycles.

The work functions of SrTiO_3_ and Cu NWs are 5.35 eV [36] and 4.65 eV [37], respectively. Due to the n-type nature and higher work function of SrTiO_3_ thin films, an ohmic contact is formed when SrTiO_3_ is interfaced with Cu NWs [38]. Figure 3a depicts the schematic band diagram for the SrTiO_3_/Cu NW interface. Upon contact, a potential barrier exists that drives a significant flow of free electrons from the Cu NWs into the SrTiO_3_. This electron transfer leads to the formation of a negative space charge region near the interface in SrTiO_3_, which in turn establishes a built-in electric field directed from SrTiO_3_ towards the Cu NWs. Concurrently, the conduction band of SrTiO_3_ bends downward while its valence band curves upward, reinforcing the n-type characteristic at the interface. Once thermodynamic equilibrium is achieved, the Fermi levels align across the interface, effectively reducing the potential barrier to zero and facilitating electron injection from the Cu NWs into the SrTiO_3_ thin films. This enhanced electron injection increases the overall carrier concentration and thereby reduces the sheet resistance in the SrTiO_3_/Cu NWs/SrTiO_3_ hybrid thin films.

For practical applications, transparent conductive films must exhibit both low sheet resistance and high light transmittance to allow maximum incident light to reach the absorber layer [39]. To optimize the optoelectronic performance of the SrTiO_3_/Cu NWs/SrTiO_3_ hybrid thin films, we evaluated the figure of merit (*Φ_TC_*) as defined by Haacke, which is calculated using the sheet resistance (*R_s_*) and transmittance (*T*) according to the following equation [40,41]:

$$\Phi_{TC}=\frac{T^{10}}{R_{s}}$$

Figure 3b shows the variation in the calculated *Φ_TC_* value for the Cu NW networks and SrTiO_3_/Cu NWs/SrTiO_3_ hybrid thin films with different spray-coating cycles. The *Φ_TC_* value increases with the number of spray-coating cycles, reaching a maximum value when the spray-coating cycle is 4. Specifically, the optimum *Φ_TC_* value of 12.35 × 10^−3^ Ω^−1^ is achieved at four spray-coating cycles, where the transmittance is 84.2% and the sheet resistance is 14.5 Ω/sq. Although a higher density of Cu NWs leads to a lower sheet resistance, further increasing the nanowire density beyond a critical threshold results in a significant reduction in optical transmittance, which in turn lowers the *Φ_TC_* value. Thus, the SrTiO_3_/Cu NWs/SrTiO_3_ hybrid thin films produced with four spray-coating cycles offer an optimal balance, ensuring enhanced light transmission in the visible range while maintaining high electrical conductivity.

**Figure 4:**
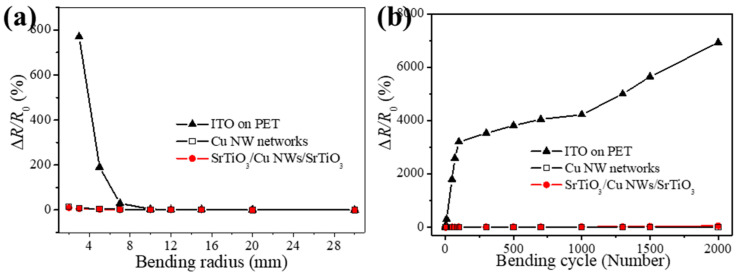
(**a**) Bending test results for the ITO on PET, SrTiO_3_/Cu NWs/SrTiO_3_ hybrid thin films, and the Cu NW networks. (**b**) Bending fatigue characteristics for the ITO on PET, SrTiO_3_/Cu NWs/SrTiO_3_ hybrid thin films, and the Cu NW networks under a constant bending radius of 5.0 mm.

Figure 4a presents the bending test results for the ITO on PET, SrTiO_3_/Cu NWs/SrTiO_3_ hybrid thin films, and the Cu NW networks. The variation in resistance is defined as Δ*R* = (*R* – *R*_0_), where *R* and *R*_0_ represent the post-bending and initial resistance, respectively. When the bending radius is reduced from 30 mm to 5 mm, the resistance of flexible ITO thin films increases dramatically by a factor of 7.7, due to the formation of cracks caused by internal stresses arising during bending. The resistance of the SrTiO_3_/Cu NWs/SrTiO_3_ hybrid thin films increases by only 5%, whereas the untreated Cu NW network exhibits a 23% increase. This suggests that the SrTiO_3_ layers serve as an effective protective coating for the Cu NW network, stabilizing the conductive pathways by mitigating the geometric slippage at the nanowire junctions. Considering that film flexibility is inversely related to thickness [42], the ultrathin SrTiO_3_ layers further contribute to maintaining the overall flexibility of the hybrid structure. Figure 4b illustrates the bending fatigue characteristics for the ITO on PET, SrTiO_3_/Cu NWs/SrTiO_3_ hybrid thin films and the Cu NW networks under a constant bending radius of 5.0 mm. After 10 bending cycles, the resistance of the SrTiO_3_/Cu NWs/SrTiO_3_ hybrid thin films increases modestly by 9% and then remains nearly constant with additional cycles, whereas the bare Cu NW network’s resistance increases by 168% after 2000 cycles. The pronounced resistance escalation in the bare network is attributed to its loosely packed structure, where bending induces significant changes at the contact junctions, leading to progressive degradation of the conductive network. In contrast, embedding the Cu NWs within the SrTiO_3_ thin films results in reinforced inter-nanowire connections, which not only minimizes initial damage due to slight nanowire breakage during the first few cycles but also stabilizes the overall resistance thereafter. These findings highlight the superior mechanical durability and adhesion of the SrTiO_3_/Cu NWs/SrTiO_3_ hybrid thin films, demonstrating their promising potential for application in flexible electronic devices where consistent electrical performance under mechanical deformation is essential.

**Figure 5:**
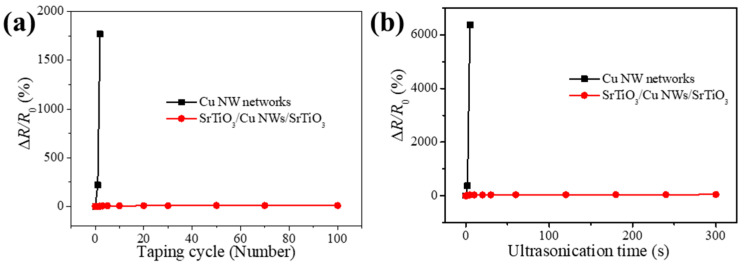
(**a**) Taping test results for the SrTiO_3_/Cu NWs/SrTiO_3_ hybrid thin films and the Cu NW networks. (**b**) Variation in resistance of SrTiO_3_/Cu NWs/SrTiO_3_ hybrid thin films and the Cu NW networks under different ultrasonic treatment durations.

Although Cu NW networks exhibit excellent conductivity and flexibility, the adhesion to substrates is poor, making them vulnerable to mechanical stresses such as tape stripping in practical applications, which severely compromises electrode stability [43,44]. To address this concern, we compared the adhesion of SrTiO_3_/Cu NWs/SrTiO_3_ hybrid thin films with that of bare Cu NW networks using 3M 610 tape tests (3M Company, USA). As shown in Figure 5a, the bare Cu NW networks lose electrical conductivity after only two tape peeling cycles, indicating that the adhesion to the PET substrate relies solely on weak van der Waals forces and is easily disrupted [45]. In contrast, the SrTiO_3_/Cu NWs/SrTiO_3_ hybrid thin films maintain stable resistance even after 100 tape peeling cycles, demonstrating that the SrTiO_3_ thin films effectively enhance the overall adhesion by embedding the Cu NWs within the thin film structure. This is because the Cu NWs are embedded between the top and bottom SrTiO_3_ thin films, and the SrTiO_3_ completely covers the Cu NW networks; thus, the SrTiO_3_/Cu NWs/SrTiO_3_ hybrid thin films show better adhesion than the pure Cu NW films. Figure 5b presents the variation in resistance of SrTiO_3_/Cu NWs/SrTiO_3_ hybrid thin films and the Cu NW networks under different ultrasonic treatment durations. The bare Cu NW networks experience a rapid increase in resistance, completely losing conductivity within one minute of ultrasonic exposure; conversely, the resistance of the hybrid thin films remains nearly unchanged under similar conditions. This indicates that the top SrTiO_3_ layers provide robust protection against ultrasonic-induced damage, effectively suppressing nanowire breakage and detachment. The addition of dense and uniform SrTiO_3_ protective layers not only significantly improves the adhesion between the Cu NWs and the substrate but also greatly enhances the stability and durability of the composite electrode in harsh environments, thereby offering a strong technical foundation for the practical application of such transparent conductive electrodes in flexible electronic devices.

**Figure 6:**
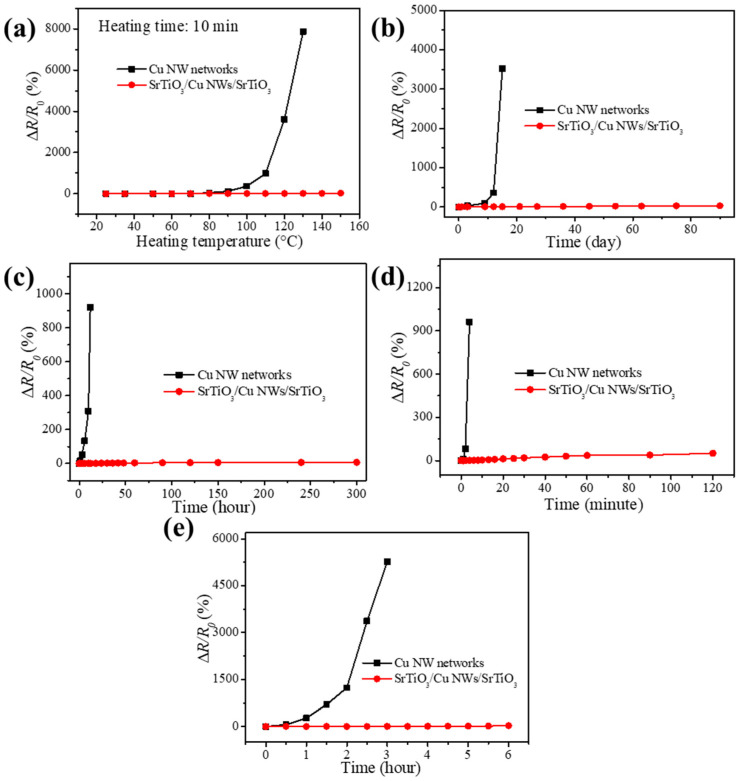
Changes in resistance of the SrTiO_3_/Cu NWs/SrTiO_3_ hybrid thin films and the Cu NW networks (**a**) with various heating temperatures and (**b**) storage times in the natural environment, (**c**) under high temperature and humidity conditions (85 °C and 85% relative humidity), (**d**) in a 3 wt% H_2_O_2_ solution, and (**e**) after UVO irradiation with different irradiation times.

The long-term stability of flexible TCFs is a critical performance parameter, particularly for applications in flexible electronic devices such as displays, wearable sensors, and thin-film solar cells that may operate in high-temperature environments [46,47]. To assess the temperature resistance of the SrTiO_3_/Cu NWs/SrTiO_3_ hybrid thin films, the samples are subjected to heating at various temperatures for a duration of 10 min, while the change in electrical resistance is continuously monitored. As illustrated in Figure 6a, at temperatures below 80 °C, both the bare Cu NW networks and the hybrid thin films maintained a constant resistance, indicating that the intrinsic electrical properties of the materials remain unaffected by thermal excitation within this range. However, when the temperature exceeds 80 °C, the resistance of the bare Cu NW networks escalates sharply, culminating in a complete loss of conductivity at 120 °C. This deterioration is primarily due to the accelerated oxidation of the Cu NWs at elevated temperatures [48], which results in the fracture of the nanowire structure and the subsequent disruption of the conductive network. In contrast, the SrTiO_3_/Cu NWs/SrTiO_3_ hybrid thin films exhibit remarkable thermal stability, with the resistance remaining virtually unchanged over the entire temperature range tested. This enhanced stability is attributed to the dense SrTiO_3_ thin films that effectively encapsulate the Cu NWs, thereby preventing the ingress of oxygen and moisture and mitigating oxidation. It should be noted that the maximum heating temperature is restricted to 150 °C due to the softening temperature of the PET substrates [49].

In addition to controlled heating tests, long-term environmental stability was evaluated by exposing the samples to ambient conditions over extended periods. As shown in Figure 6b, the bare Cu NW networks gradually undergo oxidation when exposed to atmospheric oxygen and water vapor, resulting in a progressive increase in resistance and eventual loss of conductivity after 15 days. Conversely, the SrTiO_3_/Cu NWs/SrTiO_3_ hybrid films retained their initial resistance even after 180 days of exposure, which substantiates the long-term protective effect provided by the SrTiO_3_ coatings. To further assess oxidation resistance under accelerated conditions, both the bare Cu NW networks and the SrTiO_3_/Cu NWs/SrTiO_3_ hybrid thin films are subjected to controlled tests in a high-temperature, high-humidity environment (85 °C and 85% relative humidity). Figure 6c displays the temporal evolution of resistance under these conditions. The bare Cu NW networks show a dramatic increase in resistance, ultimately losing conductivity after 18 h. This behavior is indicative of rapid oxidative degradation that compromises the nanowire structure. In comparison, the resistance of the SrTiO_3_/Cu NWs/SrTiO_3_ hybrid thin films increases by only a factor of two over the same period. The homogeneous and dense SrTiO_3_ layers function as an effective barrier, isolating the underlying Cu NWs from reactive atmospheric species. Furthermore, complementary experiments were conducted by immersing the samples in a 3 wt% H_2_O_2_ solution to simulate a chemically aggressive oxidizing environment. As depicted in Figure 6d, the resistance of the bare Cu NW networks increases sharply with immersion time, leading to a rapid deterioration of conductivity. Meanwhile, the hybrid thin films exhibit only minor resistance changes during the early stages of immersion, owing to the dense microstructure of the SrTiO_3_ layers that effectively impede the direct contact between the oxidizing solution and the Cu NWs. Although prolonged exposure eventually allows some H_2_O_2_ to permeate the SrTiO_3_ layers and initiate slow oxidation, the resultant increase in resistance remains modest compared to the bare Cu NW networks. Figure 6e shows the resistance change of the bare Cu NW and SrTiO_3_/Cu NWs/SrTiO_3_ hybrid thin films after ultraviolet ozone (UVO) irradiation. The resistance of bare Cu NWs loses the conductivity after 3 h because of the oxidation and decomposition of Cu NWs, while that of SrTiO_3_/Cu NWs/SrTiO_3_ hybrid thin films is only increased by only 26% because of the protection of SrTiO_3_ thin films.

## 4. Conclusions

This work presents the development of the high-performance flexible TCFs by using Cu NW-embedded SrTiO_3_ thin films. The conductivity of the SrTiO_3_/Cu NWs/SrTiO_3_ hybrid thin films is solely caused by the Cu NWs as the SrTiO_3_ is highly resistive. The improvement in adhesion and stability of hybrid thin films can be attributed to the effective covering of SrTiO_3_ on the Cu NW networks. Moreover, the incorporation of the SrTiO_3_ layers results in improved optical performance, with the transmittance of the Cu NW network increasing from 83.5% to 84.2% and a concurrent reduction in sheet resistance from 16.9 Ω/sq to 14.5 Ω/sq. Furthermore, when subjected to 2000 bending cycles at a curvature radius of 5.0 mm, the resistance of the hybrid thin films is increased by only 11%, thereby demonstrating exceptional mechanical flexibility. In addition, the hybrid thin films display excellent adhesion and oxidation resistance; their resistance remains unchanged even after prolonged storage in high-temperature and high-humidity environments. These outstanding properties are anticipated to facilitate the practical application and widespread adoption of SrTiO_3_/Cu NWs/SrTiO_3_ hybrid thin films in various flexible optoelectronic devices.

## Data Availability

The original contributions presented in this study are included in the article. Further inquiries can be directed at the corresponding author.
